# Directing the Way—Receptor and Chemical Targeting Strategies for Nucleic Acid Delivery

**DOI:** 10.1007/s11095-022-03385-w

**Published:** 2022-09-15

**Authors:** Ricarda Carolin Steffens, Ernst Wagner

**Affiliations:** 1 Pharmaceutical Biotechnology, Center for System-Based Drug Research, Ludwig-Maximilians-Universität, 81377 Munich, Germany; 2 Center for Nanoscience (CeNS), Ludwig-Maximilians-Universität, 81377 Munich, Germany

**Keywords:** lipoplex, pDNA, polyplex, siRNA, targeting

## Abstract

Nucleic acid therapeutics have shown great potential for the treatment of numerous diseases, such as genetic disorders, cancer and infections. Moreover, they have been successfully used as vaccines during the COVID-19 pandemic. In order to unfold full therapeutical potential, these nano agents have to overcome several barriers. Therefore, directed transport to specific tissues and cell types remains a central challenge to receive carrier systems with enhanced efficiency and desired biodistribution profiles. Active targeting strategies include receptor-targeting, mediating cellular uptake based on ligand-receptor interactions, and chemical targeting, enabling cell-specific delivery as a consequence of chemically and structurally modified carriers. With a focus on synthetic delivery systems including polyplexes, lipid-based systems such as lipoplexes and lipid nanoparticles, and direct conjugates optimized for various types of nucleic acids (DNA, mRNA, siRNA, miRNA, oligonucleotides), we highlight recent achievements, exemplified by several nucleic acid drugs on the market, and discuss challenges for targeted delivery to different organs such as brain, eye, liver, lung, spleen and muscle *in vivo*.

## Introduction

Over the last three decades, vectors for the delivery of therapeutic genetic material were extensively evaluated and optimized. Currently, more than 3000 clinical trials on gene therapy have been conducted or are still ongoing. With about 2/3 of clinical trials on cancer diseases, gene delivery to tumors represents the primary target for nucleic acid therapy. However, other indications, such as monogenetic diseases, infections, inflammatory diseases, neurological and ocular disorders are also interesting targets for gene therapy [1–3]. For gene transfer, viral vectors are still the most advanced delivery systems in clinical gene therapy studies, attributed to their excellent transduction efficacies [4, 5]. Part of their efficacy is that they are naturally built to introduce nucleic acids into foreign cells, presenting natural receptor targeting agents and peptide sequences on their surface that enable cellular entry.

**Fig. 1:**
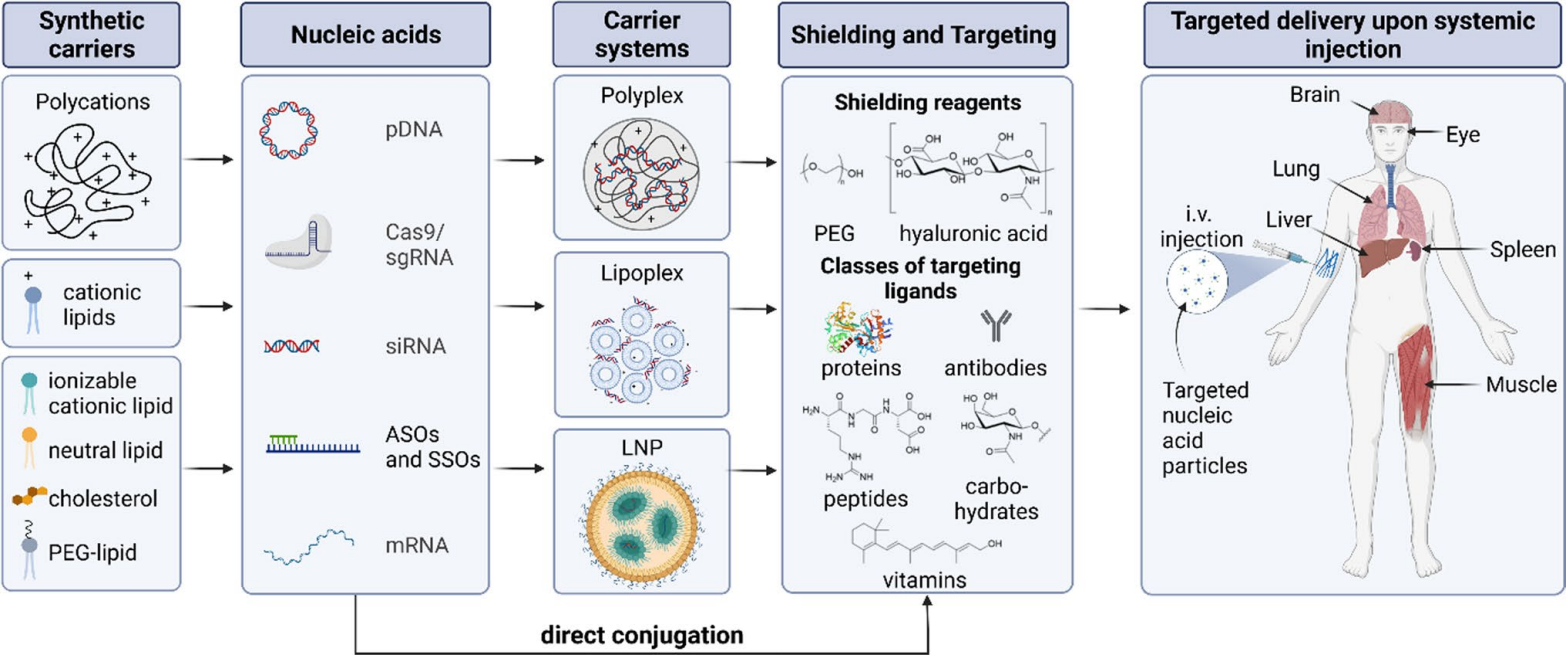
Nonviral carriers for the delivery of different nucleic acids, including their main components, particle types as well as shielding and targeting agents for organ- or cell-specific delivery upon systemic injection. Created with BioRender.com

Synthetic delivery systems present a second class of transfer agents for nucleic acid therapeutics. They have the advantage that they can be designed to be non-immunogenic and have the potential to deliver a broad range of natural or synthetic and modified nucleic acids. Historically, transfections introduced functional genes by either using DNA transfer into the nucleus or RNA transfer into the cytosol [6–8]. In contrast to DNA, messenger RNA (mRNA) does not require nuclear entry since its site of action is located in the cytosol. Its great therapeutic potential [9, 10] was recently proven by the successful application of mRNA-based COVID-19 vaccines [11–13]. With the new millennium, RNA interference (RNAi) therapeutics were developed as another class of therapeutic nucleic acids, aiming for the down regulation of malignant gene expression by short interfering RNA (siRNA) [14] or micro-RNA (miRNA) [15]. In addition, now for more than 30 years, antisense oligonucleotides have been therapeutically applied by blocking or modulating splicing of complementary mRNA [16, 17]. Most recently, the CRISPR/Cas technology has entered therapeutic application as a promising tool for genome editing [18, 19], either *via* Cas9 mRNA/single guide (sg) RNA or as Cas9 protein/sgRNA ribonucleoprotein complex [20]. These synthetic carriers range from organic, polycationic carriers to lipid structures and inorganic particles and were extensively optimized in order to enhance transfection efficacy and become more and more like artificial viruses [21, 22]. First of all, these synthetic carriers are designed to compact the genetic material for protection against degradation and shielding against undesired interactions. This can be achieved by polycations such as poly-L-lysine (PLL) [23, 24], polyethylenimine (PEI) [25, 26], poly(amidoamines) (PAMAM) or dendrimers [27, 28] which interact with negatively charged nucleic acids and assemble in nano-sized particles, so-called “polyplexes”[29]. Lipid-based structures including “lipoplexes”, in which the genetic material is packaged by positively charged lipidic carriers, and “LNPs”, containing the nucleic acid inside multi-component lipid nanoparticles, have also proven great potential as nucleic acid delivery systems (Fig. 1) [29, 30].

In order to show comparable transfection efficacies to viral vectors, especially for *in vivo* studies, synthetic delivery systems need to fulfill further demands. The particles should a) show no interaction with blood components or aggregation tendency in physiological environment, b) show prolonged blood circulation time to reach the target tissue, c) promote efficient cellular uptake and d) release the genetic material into the cytoplasm, so it can reach its site of action.

Within this delivery process, one major challenge remains the transport and transfer of nucleic acids to the desired cell type or tissue. This requirement can be approached by modifying synthetic carriers with functional domains giving targeted delivery systems, which was first successfully implemented 35 years ago by Wu *et al*. using GalNAc-presenting ligands on PLL-polyplexes for directed delivery to hepatocytes [24, 31].

In this review, we will give an overview about different active targeting strategies for synthetic delivery systems. We highlight recent advances in nucleic acid delivery to specific healthy tissues including the liver, lung, brain, immune cells, retina and muscle. Specific delivery to cell types can be mediated *via* defined ligand-receptor interactions (*receptor-targeting*) as well as modulating the physicochemical properties of the nucleic acid nanoparticles based on small structural variations of the synthetic carriers (*chemical targeting*). For tumor-specific targeting of nucleic acids we refer to other published work [32–34].

## Strategies for specific delivery

### Shielding

Synthetic carriers have proven to be potent transfer vehicles for nucleic acid delivery for *in vitro* studies thanks to various optimizations. However, during the delivery process from injection to gene expression, several obstacles and cellular bottlenecks must be overcome to unfold the full therapeutic potential of the nucleic acid. Besides efficient encapsulation of the genetic material to protect against degradation, it must be ensured that the carriers circulate in the blood until reaching the target tissue.

**Fig. 2:**
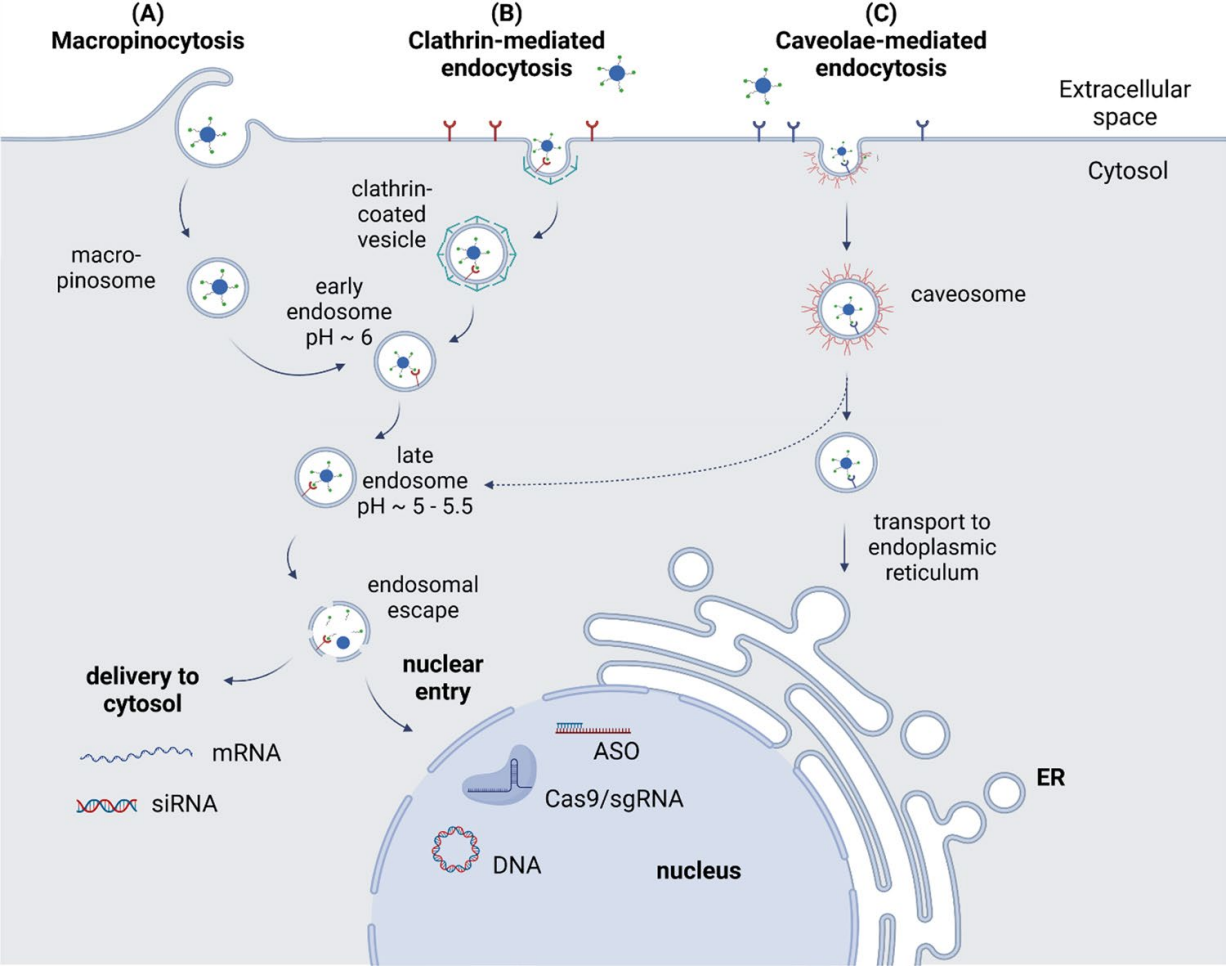
Internalization pathways of nucleic acid carriers by receptor-mediated endocytosis. Created with BioRender.com

Cellular uptake of positively charged polyplexes is enabled by non-specific endocytosis, in particular macropinocytosis from the extracellular fluid [35] (see Fig. 2A). The uptake can be further improved, even though not specified, by incorporation of cell-penetrating peptides in the formulation, such as octaarginine motifs [36, 37]. *In vivo* applied nanocarriers, however, are confronted with blood components like plasma proteins that adsorb on particle surface and hence sustainably affect circulation, transport to tissues and cellular uptake [38–40]. For instance, the formation of a protein corona comprised of opsonins will mediate phagocytosis removing particles from the circulation.

To this end, it is necessary to shield positively charged carriers against unspecific interactions with serum proteins that may result in phagocytosis of the particles. This can be achieved by incorporating shielding domains, for example polymers such as polyethylene glycol (PEG) [41], poly(N-(2- hydroxypropyl)methacrylamide) (pHPMA) [42], poly(2-oxazolines) [43] and polysarcosines [44] or polysaccharides such as hydroxyethyl starch [45] or hyaluronic acid (HA) [46–48] (Fig. 1). Shielding the carrier’s surface results in lowered surface charge and thus reduced interaction with serum proteins, which allows the particles to circumvent the reticuloendothelial system (RES) and increase circulation time in the blood [41]. However, poor targeting abilities resulting in off-target effects or low accumulation on the target site set limitations regarding the application of synthetic gene delivery systems *in vivo*. Consequently, to ensure tissue- or cell-selective delivery and to minimize accumulation in off-target sites, the particles can be surface-modified either by specific ligands that will interact with receptors on the targeted cell type for active targeting or by modification of their chemical composition leading to altered biodistribution for chemical targeting.

### Active Targeting

#### Receptor Targeting

Decorating the carrier’s surface with ligands can yield specific cellular uptake based on receptor-mediated endocytosis. Here, the fact that specific tissues differently express certain receptors is used to enhance cell-specific uptake of nucleic acid carriers [49]. The types of ligands used for modification of delivery systems range from small chemical drug-derived compounds [50–53], peptides [54, 55] to large proteins [56, 57], antibodies [58, 59], carbohydrates [60–62] and vitamins [63, 64] **(**Fig. 1). Interaction with their specific receptors will lead to receptor-mediated endocytosis, a highly selective process of nanoparticle internalization.

In order to achieve receptor-mediated uptake, unspecific interactions of the carrier with blood-components have to be reduced by shielding. Cellular uptake is initiated by recognition of specific ligands by cell surface receptors. After cell binding, the receptor-ligand complex is internalized by the formation of vesicles from the cell membrane and delivered to the cytosol (Fig. 2B, C). The fate of nanocarrier transport through the cytosol is determined by the receptor type and the associated pathway of endocytosis, as summarized in Table I. For example, prominent receptors used for targeted gene delivery such as transferrin receptor (TfR) [65], asialoglycoprotein receptor (ASGPR) [66] or low-density lipoprotein receptor (LDLR) [67] undergo clathrin-mediated endocytosis into early endosomes, followed by endosome maturation and fusion with lysosomes [68]. Only small particles with a maximum size of 200 nm can be taken up by this route [69]. Other receptors, e.g., the folate receptor [70] or interleukin-2 receptor [71], are internalized *via* caveolae-mediated endocytosis [72]. This route tolerates uptake of particles up to 500 nm [69] and allows to bypass the fusion with lysosomes, leading to delivery to endoplasmic reticulum and facilitated nuclear transport [73]. Generally, the release of carriers from late endosomes is critical for efficient nucleic acid delivery to avoid either enzymatic degradation of the nucleic acid payload after fusion with lysosomes or exocytosis during receptor recycling. Conveniently, synthetic carriers have been designed to exhibit endosomolytic properties. For example, cationic carriers containing protonatable amines, e.g., PEI or histidine-containing constructs response to acidic pH in late endosomes promoting a proton-sponge effect, i.e. swelling and eventually disruption of the endosomes, leading to release of the nucleic acid to the cytosol [74, 75]. Lipid-based carriers are able to release their cargo by fusion with the negatively charged endosome membrane [76].

**Table I:** Characteristics of Cellular Uptake via Clathrin- and Caveolae-Mediated Endocytosis

	Clathrin-mediated endocytosis	Caveolae-mediated endocytosis
Vesicles for transport	Clathrin-coated vesicle	Caveosome
Examples for receptors	TfR, ASGPR, LDLR	Folate receptor, VEGFR, Interleukin-2
Tolerated nanoparticle size	Up to 200 nm	200 to 500 nm
Intracellular fate of cargo	Formation of endosomes, maturation to late endosomes, lysosomal degradation or endosomal escape	Transport to endoplasmic reticulum, Golgi apparatus, facilitated delivery to nucleus

#### Strategies to Incorporate Targeting Ligands into the Delivery System

Ligands for receptor-targeting as well as shielding domains can be integrated into the delivery system by both, pre-functionalization, and post-modification [77]. Multivalent ligand presentation on the carrier’s surface may promote receptor recognition and increases binding affinity [78, 79]. The density of ligands required for efficient targeting strongly depends on both the chosen carrier system as well as the type and avidity of ligand. For polyplexes, the ligand to polycation ratio may range from 2.5% to > 100% functionalization, depending on the type of ligand [80–82]. In LNPs, ligand-functionalized lipids may account for only 1–2 mol% per formulation, but still promote target-specific delivery [83, 84]. In direct conjugates such as trivalent GalNAc-siRNA, every nucleic acid is equipped with a targeting moiety.

Accessibility of the targeting ligand is also important for receptor binding [85].

Pre-functionalization has been evaluated for polymeric delivery systems which contain different domains for nucleic acid binding, shielding, and targeting [62, 86, 87]. An alternative functionalization strategy is based on the post-modification of pre-formed nanoparticles, mostly *via* covalent attachment of ligands to functional groups displayed on the surface. For example, copper-free [88, 89] or copper(I)-catalyzed [90] alkyne-azide click reactions were used for particle modification, as they are fast, selective and high-yielding. By means of this method, it can be ensured that the ligands are located on the surface of the particles. At the same time, the removal of excess ligands that may not have bound to the carrier may be required, as they could compete for the receptor and reduce cellular uptake.

Non-covalent binding of ligands to the particle surface can also be considered. This modification method was successfully realized for targeting of synthetic carriers with insulin [91] and hyaluronic acid [48].

#### Dual Targeting

Inspired by natural viruses, which have optimized cell association and cellular entry mechanisms by presenting several ligands on their surface, dual targeting represents an approach to further increase transfection efficacy. Here, cell entry properties of viral vectors are mimicked by using two (or more) ligands on a single carrier. For example, Nie *et al*. used dual-targeted PEGylated PEI-pDNA polyplexes, modified with the cell binding peptide B6 and the integrin targeting peptide RGD for increased transfection efficiency on DU145 and PC3 cells, showing increased transfection efficacy when both ligands were incorporated in the polyplex formulation. In addition, it was demonstrated how these ligands participate in both, cell association and internalization [92]. Additional studies of dual-targeted polyplexes with combinations of B6, GE11 (for EGFR targeting) and cyclic cRGDfk (for integrin targeting), respectively, revealed that the combination of B6 and GE11 was most promising for pDNA transfections to DU145 cells, which express all three receptors [93]. Dual-targeted LPEI-PEG polyplexes were also used for delivering the theranostic sodium iodide symporter gene to Huh7 cells, using a combination of GE11 and cMBP, which showed strong benefits compared to single-targeted polyplexes [94].

#### Cascade Targeting

Under certain circumstances, nucleic acid carriers have to overcome several barriers to reach their site of action, e.g., the blood–brain-barrier (BBB), followed by membranes of targeted cells. In order to generate cascade targeting delivery systems, nanoparticles can be designed to cross the BBB first and display targeting ligands selectively binding to receptors on specific cells behind the barrier. For example, Wang *et al*. developed such a gene delivery system by decorating the carrier with the I_6_P_7_ ligand, a heptapeptide derived from interleukin-6, which is able to promote both, BBB crossing and cell-specific delivery to interleukin-6 receptor presenting cells [95]. A cascade targeting concept was also used by Zhang *et al*. to deliver siRNA into neuronal cells after crossing the BBB for treatment of Alzheimer’s disease. In this study, BBB crossing was achieved *via* the ApoA-I ligand, which binds to the scavenger receptor B1, and selective uptake of the particles by neuronal PC12 cells could be demonstrated by incorporation of a peptide ligand, NL4 binding to tropomyosin receptor kinase A (TrkA). These findings were subsequently confirmed by *in vivo* studies resulting in downregulation of BACE1, an enzyme which is involved in pathogenesis of Alzheimer’s disease [96, 97].

#### Chemical Targeting

In addition to actively targeted delivery supported by ligand-receptor specific interaction, chemical properties of the carrier system can also generate cell- or tissue-specific delivery. Unmodified polycationic carriers such as poly-L-lysine or PEI naturally interact with negatively charged heparan sulfate proteoglycans of the plasma membrane which leads to particle uptake [98, 99]. Lipoplexes and liposomes containing cationic lipids were found to destabilize the phospholipid bilayer of cell membranes and are subsequently internalized *via* receptor-independent endocytosis [100]. Recently, it was observed that liposomes and LNPs typically accumulate in the liver in their classical composition due to non-covalent attachment of serum proteins, especially apolipoprotein E (ApoE) resulting in transport to hepatocytes and uptake *via* low-density lipoprotein receptor (LDLR) [101–104]. In fact, the composition of the carrier systems largely influences the interaction with blood proteins [105]. Therefore, together with active targeting for tissue- or cell-specific nucleic acid delivery by ligand-receptor specific interaction, chemical adjustment of the carrier system can be also utilized to generate organ-specific delivery. It could be observed that slight changes of the chemical or physicochemical properties of the formulation such as particle size and surface charge have a remarkable impact on the biodistribution and accumulation in certain tissues or cell types.

Chemical targeting has shown great potential for ligand-independent, but yet organ-specific delivery of nucleic acids. For example, Kowalski *et al*. observed organ-selective distribution of mRNA-LNPs with a library of amino-polyesters either in liver, spleen, or lung after i.v. injection [106]. Localization of structurally different LNPs after systemic administration was investigated by Dahlman *et al*. by developing a barcode tool in order to track biodistribution *in vivo* [107]. Furthermore, the group of Siegwart synthesized libraries of lipids for LNP formulations for delivery of mRNA as well as Cas9 with different properties by variation of charge, hydrophobicity and pKa, respectively, resulting in so-called “selective organ targeting” (SORT) lipids and studied the accumulation of these formulations in different tissues and cell types. For example, it was demonstrated that particles with higher amounts of positively charged dioleoyl-3-trimethylammonium propane (DOTAP) preferably accumulated in the lung, whereas the addition of negatively charged lipids led to selective delivery to the spleen [108, 109]. Recently, the mechanism behind SORT was studied, revealing both global and apparent pKa as well as the composition of the serum protein corona of the LNP formulation determining the selective delivery. The study revealed first that particles with a pKa around 6–7 accumulated in the lung, while LNPs with lower pKa from 2 to 6 were preferably delivered to the spleen and second that different serum proteins bind to LNPs dependent on the component composition, therefore representing pioneering results for understanding LNP delivery beyond hepatocytes for future fields of applications [110].

## Liver as Target

The liver is a highly metabolic organ and source of numerous expressed genes and plasma proteins. Not surprisingly, this organ is also a main target for nucleic acid and gene therapy of a series of severe hereditary monogenetic diseases [111–113]. In addition, non-inherited hepatic diseases such as liver cirrhosis or hepatitis B and C or hepatocarcinoma are life-threatening [114]. Therefore, the liver presents a high-priority target for nucleic acid delivery. Targeting of hepatocytes can be approached either by active or indirect active targeting, dependent from the carrier system as summarized in Table II.

**Table II:** Targeted Nucleic Acid Delivery to Different Liver Cell Types

	Receptor	Delivery system	Ligand	Type of nucleic acid	Results	Reference
Hepatocytes						
	ASGPR	PLL polyplex	ASOR	pDNA	First report on targeted, hepatocyte-specific gene delivery	[24, 31]
		PLL polyplex	Artificial tetra-antennary GalNAc ligand	pDNA	Conjugation of artificial ligand to the polyplexes results in comparable gene transfer efficiency as with the natural ligand asialofetuin, monovalent ligand does not improve gene expression	[60]
		Polymer-nucleic acid conjugate	GalNAc	siRNA	*in vitro* and *in vivo* hepatocyte-specific delivery of siRNA	[120]
		Direct conjugate	Tri-GalNAc	siRNA	FDA and EMA approval of several products:	
		Direct conjugate	Tri-GalNAc	siRNA	-Givosiran for treatment of acute intermittent porphyria	[123]
		Direct conjugate	Tri-GalNAc	siRNA	-Lumarisan for treatment of primary hyperoxaluria type 1	[124, 125]
		Direct conjugate	Tri-GalNAc	siRNA	-Inclisiran for treatment of primary hypercholesterolaemia	[128]
		LNP	Tri-GalNAc	siRNA	Exogenous ligand Tri-GalNAc mediates ASGPR-dependent uptake	[104]
		Direct conjugate	Tri-GalNAc	ASO	Enhanced uptake, improved delivery and activity duration of clinically relevant ASOs to hepatocytes *in vivo*	[130]
		Direct conjugate	Tri-GalNAc	ASO	Improved uptake and activity of targeted ASOs in human clinical trial	[133]
		Direct conjugate	Tri-GalNAc	ASO	Safety, pharmacokinetic and pharmacodynamic study of GalNAc-ASO for treatment of β-thalassemia in monkeys	[134]
		Direct conjugate	Tri-GalNAc	ASO	Enhanced uptake by hepatocytes, but not other liver cell types	[131]
		Direct conjugate	Tri-GalNAc	ASO	Increased delivery of anti-miRNA-ASOs to hepatocyte in presence of ligand	[132]
		Direct conjugate	Tri-GalNAc	Cas9 RNP	Disulfide linkage between Cas9 and GalNAc ligand led to receptor-dependent, selective uptake by hepatocytes and exhibited gene editing activity	[135]
	LDLR	Direct conjugate	ApoB (endogenous)	siRNA	Delivery of siRNA to hepatocytes generated gene silencing of apoB protein expression	[136]
		Direct conjugate	AopB (endogenous)	ASO	Improved uptake of ASOs by hepatocytes after ligation of cholesterol	[131]
		LNP	ApoE (endogenous)	siRNA	Uptake of LNPs is mediated by LDL-receptor determined by formation of ApoE-containing protein corona	[104]
		LNP	ApoE (endogenous)	siRNA	Development of Patisiran for treatment of hereditary transthyretin amyloidosis, EMA and FDA approval in 2018	[137]
		LNP	ApoE	Cas9-mRNA/ sgRNA	Efficient TTR gene knockout *in vivo* in phase 1 clinical trial (57% after infusion of 0.1 mg/kg and 87% after 0.3 mg/kg); mild adverse effects	[19]
Hepatic stellate cells						
	RBP receptor	Liposome	Vitamin A	siRNA	Down-regulation of collagen synthesis after RBP receptor mediated uptake led to resolution of liver cirrhosis and fibrosis in rats after repeated treatments	[64, 141]
	PDGFR β	LNP	Cyclic peptide pPB	siRNA	Increased uptake of targeted SNALPs by HSCs; accumulation in liver after i.v. injection in mice, down regulation of gp46 mRNA expression, which is high in hepatic fibrosis	[83]
Liver sinusoidal endothelial cells and Kupffer cells						
	n.a	LNP	None (chemical targeting)	Barcode DNA, siRNA, sgRNA, mRNA	Alteration of cholesterol in LNP composition: Oxidized and esterified cholesterol mediated uptake by LSECs, cholesterol-oleate led to threefold enhanced gene editing activity in LSECs compared to hepatocytes	[143, 144]
	n.a	LNP	None (chemical targeting)	Barcode DNA, mRNA	Exchange of DLin-MC3 by cKK-E12 leads partly to LNP uptake by LSECs and KCs	[142]
	n.a	LNP	None (chemical targeting)	Barcode DNA, mRNA	Adamantyl-phospholipids shifted distribution from hepatocytes to KCS and LSECs, but not to extrahepatic immune cells	[145]
	n.a	LNP	None (chemical targeting)	mRNA	Uptake by LSECs and KCs was achieved by replacing zwitterionic DSPC with anionic DSPG	[146]

Abbreviations: ASGPR, Asialoglycoprotein receptor; PLL, poly-L-lysine; ASOR, asialoorosomucoid; GalNAc, *N*-acetyl galactosamine; FDA, U.S. Food and Drug Administration; EMA, European medicines agency; LDLR, low-density lipoprotein receptor; ApoB, apolipoprotein B; ApoE, apolipoprotein E; RBP, retinol binding protein; PDGFR β, platelet-derived growth factor receptor β; pPB; SNALPs, stable nucleic acid lipid particle; HSC, hepatic stellate cell, LNP, lipid nanoparticle; LSECs, liver sinusoidal endothelial cells; DLin-MC3

### Hepatocytes

#### Hepatocyte Targeting *via* Asialoglycoprotein Receptor

Delivering nucleic acids into hepatocytes is mostly achieved by receptor-mediated endocytosis *via* the ASGPR, which is found almost exclusively and abundantly on hepatocytes [115]. ASGPR binds highly selective to terminal, multi-antennary galactose and N-acetyl galactosamine (GalNAc) residues of glycoproteins with defined spatial geometry in presence of calcium(II)-ions [116–118].

The first targeted delivery of DNA by a non-viral delivery system was reported by Wu *et al*. by using asialoorosomucoid-modified (ASOR) polylysine polyplexes mediating delivery to the liver *via* ASGPR *in vitro* and *in vivo* after intravenous (i.v.) injection [24, 31]. Later, Plank *et al*. used an artificial tetra-antennary galactose ligand for gene transfer of pDNA-poly(lysine) polyplexes to hepatocytes *in vitro* [60]. Artificial ligands for ASGPR targeting were extensively optimized, as spatial distance of the carbohydrate residues, a well-balanced equilibrium of hydrophilicity and hydrophobicity of the linker largely impact the binding affinity towards the receptor [119].

Rozema *et al*. developed a dynamic polymer-nucleic acid conjugate, that fulfilled several tasks in one: The backbone itself provided endosomolytic properties, served as reaction site for the covalent attachment of siRNA and was grafted with GalNAc and PEG, giving a targeted and shielded vehicle. This formulation induced gene silencing activity in the liver after i.v. injection in mice [120].

**Fig.3:**
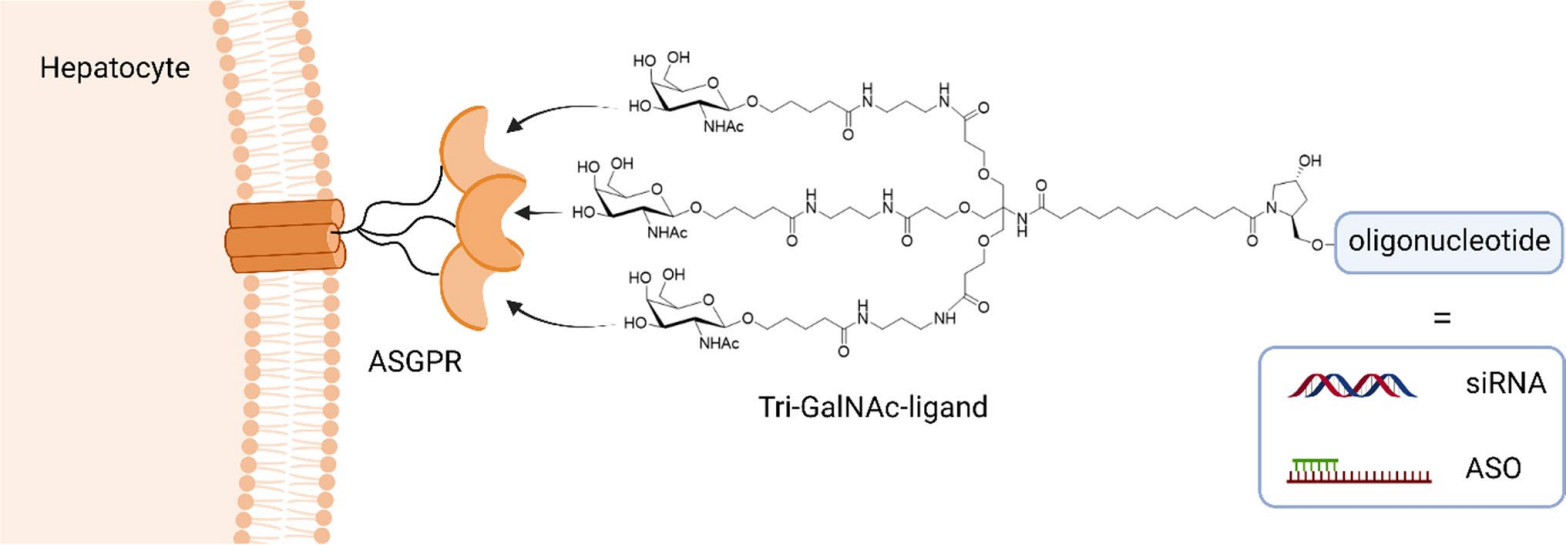
Optimized trivalent GalNAc-ligand for hepatocyte delivery of direct conjugates with siRNA and ASOs, respectively, via ASGPR-mediated endocytosis. Created with BioRender.com

One milestone in both RNAi therapeutics and ASGPR mediated nucleic acid delivery is represented by the market release of Givosiran (Givlaari) in 2019 by the U.S. Food and Drug Administration (FDA) and European medicines agency (EMA). The nucleic acid, a chemically modified and stabilized siRNA, is directly conjugated to a tri-GalNAc ligand optimized towards ASGPR binding sites (Fig. 3). When administered subcutaneously, the direct conjugate enabled efficient gene silencing of aminolevulinic acid synthase 1 (ALAS1) aiming for reduced levels of ALA and PBG metabolites by RNAi for the treatment of acute hepatic porphyria [121–123]. Since then, two additional RNAi-therapeutics have been released to the market for treating rare, monogenetic hepatic diseases, using the same concept, i.e., the direct conjugation of trivalent GalNAc ligand to therapeutic siRNA. Lumasiran (Oxlumo) gained FDA approval in 2020 and targets the silencing of the gene encoding glycolate oxidase for the treatment of primary hyperoxaluria type 1 (PH1) [124, 125]. Inclisiran (Leqvio) also represents a direct siRNA-TriGalNAc conjugate targeting the inhibition of the translation of serum protease PCSK9 in order to regulate cholesterol blood level for the treatment of hypercholesteremia after subcutaneous injection. [126–128]. Thanks to the success of GalNAc-siRNA direct conjugates, several further therapeutics based on the same concept are currently in phase 2/3 clinical trials [129].

Additionally, the approach of direct conjugates has been further expanded to other cargos, e.g., antisense oligonucleotides [130–134] or Cas9 RNP complexes [135]. For example, trivalent GalNAc ligands were conjugated to antisense oligonucleotides, which enabled hepatocyte-specific delivery and enhanced the activity of clinically relevant human ASOs in mouse models [130], monkeys [134] and humans [133]. In another study, ASOs were conjugated with trivalent GalNAc and cholesterol, respectively, reporting enhanced uptake by hepatocytes [131]. Recently, Yamamoto *et al*. demonstrated that the conjugation of GalNAc to anti-miRNA ASOs led to highly increased potency [132]. A novel, trivalent GalNAc ligand which showed high affinity towards ASGPR (K_D_ < 100 pM) mediated receptor-dependent, hepatocyte specific delivery and selective gene editing of CRISPR/Cas9 RNP complex [135].

#### Hepatocyte Targeting *via* LDL Receptor

In addition to active targeting of hepatocytes *via* ASGPR by GalNAc-modified formulations, uptake of lipid formulations can be also achieved by LDLR-mediated endocytosis. For example, cholesterol-siRNA direct conjugates for apolipoprotein B (ApoB) silencing have been found to exhibit gene silencing activity in hepatocytes *in vivo* [136]. LDLR-mediated uptake was observed due to interactions of cholesterol with serum proteins. The same approach was used for ASO direct conjugates by Watanabe *et al*. to enhance uptake and ASO activity in hepatocytes [131].

Furthermore, it was found that neutral liposomes interact mostly with apolipoprotein E (ApoE) in the blood, which directs the transport to hepatocytes by LDLR-mediated endocytosis [101–103]. Based on this observation, it was concluded that LNPs, multicomponent mixtures of cholesterol, an ionizable, cationic lipid, neutral helper lipids and a PEG-lipid for nucleic acid compaction, which appear almost neutral in serum, interact in a similar way with ApoE [104]. Thus, ApoE was identified as an endogenous ligand mediating the hepatocytic uptake of LNPs *via* LDLR [104].

In particular, Patisiran (Onpattro), the first siRNA product which was approved by FDA and EMA in 2018, is using LDLR interaction for the delivery of siRNA against transthyretin (TTR) mRNA to treat hereditary TTR mediated amyloidosis. By using a sheddable PEG-component, the formation of ApoE protein corona was observed, resulting in hepatocyte uptake *via* LDL receptor [137, 138]. In a recent phase 1 clinical study by Gilmore *et al*. the therapeutic effect of Cas9-mRNA/sgRNA targeting TTR, encapsulated by LNPs was evaluated. As a result of efficient TTR gene knockout, an average decrease of 87% of TTR protein levels was observed after one month in the patient group that received 0.3 mg/kg, accompanied with only mild side-effects. [19].

## Targeting Liver Cell Types Beyond Hepatocytes

**Fig. 4:**
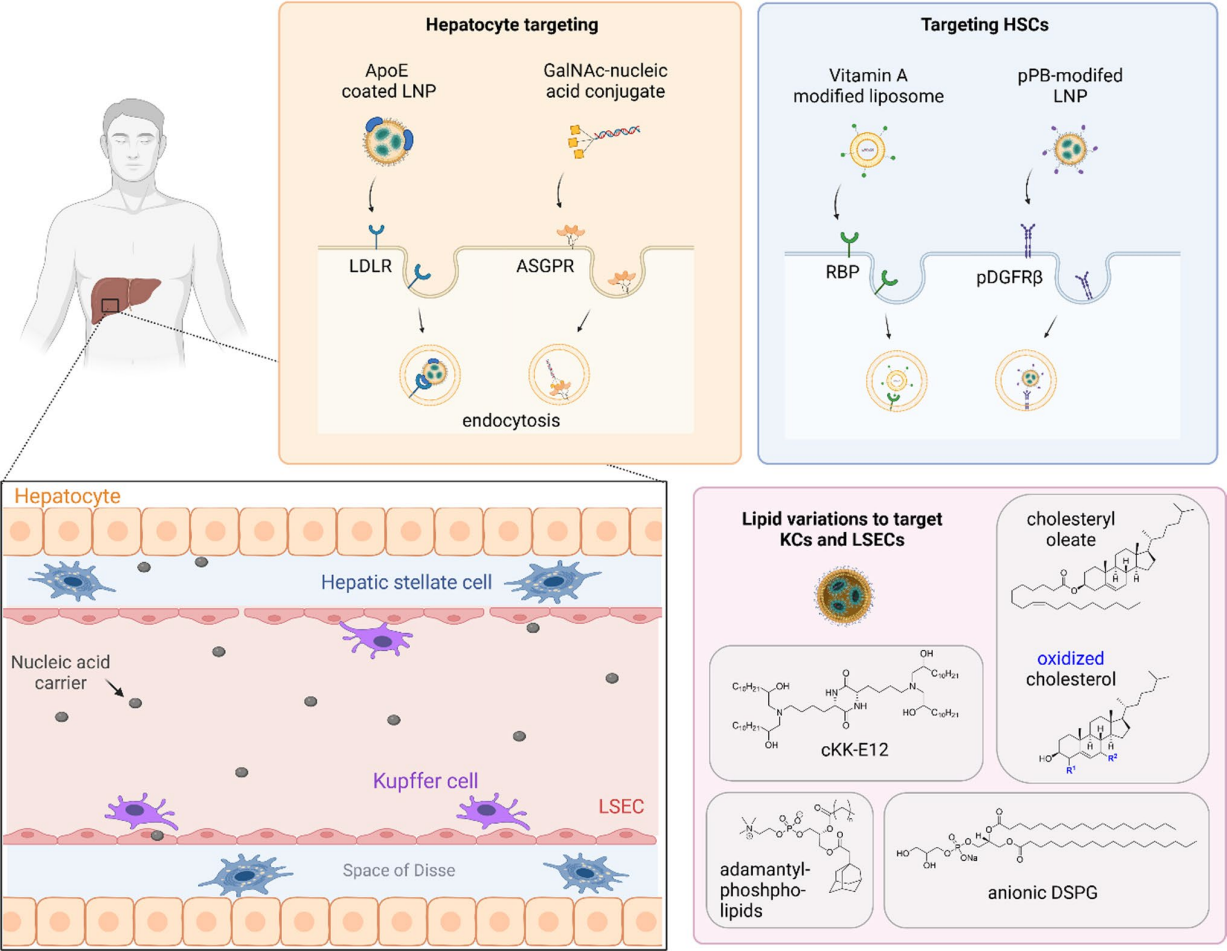
Strategies to target different liver cell types: Hepatocytes (orange), hepatic stellate cells (blue), Kupffer cells (purple) and liver sinusoidal endothelial cells (light red). Created with BioRender.com

Approximately 80% of the liver is composed of hepatocytes. However, other cell types which are part of the hepatic reticuloendothelial system (RES), such as liver sinusoidal endothelial cells (LSECs), hepatic stellate cells (HSCs) and Kupffer cells (KCs) also represent interesting targets for nucleic acid therapeutics. Although KCs are very effective in removing and destroying nanosystems, they are much more difficult to be productively transfected with commonly used nucleic acid carriers. Therefore, several attempts were made to enable nucleic acid delivery to these cell types, including receptor-targeting strategies as well as the development of novel lipids for LNPs aiming for chemical targeting (Fig. 4).

### Hepatic Stellate Cells

While hepatic stellate cells make up about 5–8% of the cells in a healthy liver, the fibrotic liver consists of 15% HSCs. Nucleic acid delivery to activated HSCs is believed to reduce fibrosis by regulating fibrogenic cytokines [139, 140].

For example, Sato *et al*. accomplished HSC-targeted delivery of liposomes and LNPs by decorating the particle surface with vitamin A [64, 141]. As HSCs are a main storage for vitamin A, uptake of the liposomes was mediated by retinol binding receptor, which led to suppression of cirrhosis in a cirrhotic liver rat model by delivering therapeutic siRNA [64] and showed ability to promote regeneration of chronically injured liver [141]. Fibrotic HSCs were moreover targeted by modification of siRNA-LNPs with a cyclic peptide ligand (pPB) that interacts with the platelet-derived growth factor receptor β. It could be observed that pPB-targeted LNPs accumulated with high specificity in HSCs confirmed by biodistribution experiments after systemic injection [83].

Studies conducted by the Dahlman group using the barcode technology described before for *in vivo* screening of lipid compositions aimed for delivery of chemically different LNP formulations beyond hepatocytes and the understanding of particle distribution in the liver microenvironment without the requirement for additional receptor-targeting ligands [142].

### Liver Sinusoidal Endothelial Cells and Kupffer Cells

Targeted nucleic acid delivery to LSECs and KCs, which belong to the hepatic RES using chemical targeting has moved into the focus of attention.

Dahlman *et al*. used their barcode screening tool to tune the LNP biodistribution in mice based on alterations in the cholesterol component. As a consequence, the biodistribution shifted from hepatocytes to LSECs and KCs, respectively, using cholesterol-oleate or oxidized cholesterol [143, 144]. Additionally, the distribution of commercial LNP formulations with DLin-MC3 compared to the ionizable lipid cKK-E12 was assessed, revealing that both formulations were not only delivered to hepatocytes but partly to LSECs and KCs [142]. Furthermore, variations within the alkyl chains of the phospholipids giving “constrained” adamantyl-phospholipids which delivered the cargo specifically to KCs and LSECs, but not to immune cells outside the liver were evaluated [145].

Recently, Pattipeiluhu *et al*. developed LNPs for delivery of mRNA to hepatic RES resembling the Onpattro® formulation. By replacing the phospholipid within the LNP from the zwitterionic 1,2-distearoyl-sn-glycero-3-phosphocholine (DSPC) to the anionic 1,2-distearoyl-sn-glycero-3-phosphoglycerol (DSPG) a negative surface charge was created and specific uptake by LSECs under participation of Stabilin receptors in embryonic zebrafish and mice was achieved [146].

## Immune Cells as Targets

Immune cells represent an interesting target for nucleic acid delivery as they play an important role in a wide range of diseases, including cancer, inflammatory or autoimmune diseases, etc. Besides the hepatic RES, immune cells are ubiquitous in the organism, especially in the spleen. Delivery of nucleic acid therapeutics to leukocytes, which include macrophages and dendritic cells as well as lymphocytes, offers the way to introduce genetic material with anti-inflammatory potential or to provoke T-cell modulation as a mean of immune stimulation [147, 148].

### Macrophages

Macrophages, including the aforementioned Kupffer cells, express membrane lectins which recognize certain carbohydrate patterns, such as the mannose receptor CD206, that mediate endocytosis as a central function of immune response [149, 150]. This mechanism was used for targeted delivery of nucleic acids to macrophages. For example, DNA transfection to macrophages was accomplished by Erbacher *et al*. using mannosylated PLL polyplexes by interaction with the mannose receptor [151]. Mannosylation of PEG-PLL polyplexes caused an increase in transfection efficacy by about 8 times compared to untargeted polyplexes in a recent study by Lopukhov. Within the same study, the transfection efficacy of polyAsp(DET)-DNA polyplexes was boosted about 500 times when mannose residues were incorporated in the formulation [152]. Moreover, mannose-functionalized nanohydrogels have shown to efficiently deliver siRNA to CD206 + primary macrophages both *in vitro* and *in vivo*, which offers the opportunity for targeted gene regulation in immunosuppressive macrophages [153, 154]. In addition, Uehara *et al*. demonstrated efficient, ligand dependent gene silencing activity of a direct conjugate between siRNA and a tetravalent, chemically modified mannose in macrophages, which represents the first report of systemic delivery of siRNA-ligand conjugates to leukocytes [61].

### Dendritic Cells

DCs, serving as antigen presenting cells (APCs), play a crucial role in antigen recognition of antigens and activation of immune response after uptake of foreign particles. Particles are internalized *via* phagocytosis or receptor-mediated endocytosis, dependent on their size and surface modifications. However, for immunotherapy it can be desirable to target DCs specifically (Table III).

**Table III:** Active Targeting of Different Immune Cell Types

	Receptor	Ligand	Delivery System	Type of nucleic acid	Key results	Reference
Macrophages						
	Mannose receptor	Mannose	PLL polyplexes	DNA	Transfection to monocyte-derived macrophages using Man-PLL polyplexes	[151]
		Mannose	PLL polyplexes and pAsp(DET) polyplexes	DNA	8× increased transfection efficacy for Man-PLL polyplexes, 500× increased transfection efficacy for Man-pAsp(DET) polyplexes in murine bone marrow derived macrophages	[152]
		CM Mannose	Direct conjugate	siRNA	Ligand-dependent gene silencing in monocyte-derived macrophages (*in vitro*) and in splenic and liver macrophages (*in vivo*)	[61]
		Mannose	Cationic nano-hydrogel	siRNA	Receptor-dependent delivery of siRNA to M2 macrophages and efficient gene knockdown in primary cells and in mice	[153, 154]
Dendritic cells						
	Mannose receptor	Mannose	PEI polyplexes	pDNA	Increase of transfection efficacy by mannosylation of PEI polyplexes, uptake was reduced in presence of the inhibitor Mannose-BSA	[82]
		Mannose	PEGylated LNPs	mRNA	Variation of PEG-spacer length (PEG100, PEG1000 and PEG2000) was evaluated; LNPs with Man-PEG1000 showed highest transfection efficacy	[156]
		CMM	Direct conjugate	siRNA	Ligand-dependent gene silencing activity in monocyte derived DCs	[61]
		Mannan	LNP	Self-amplifying RNA	Enhanced immunization was observed for LNPs decorated with multivalent mannose residues	[157]
	DEC205	Anti-DEC205 scFv	LNP	siRNA	DEC205-dependency on uptake was demonstrated; targeted LNPs showed twofold increase in uptake compared to untargeted LNPs and LNPs with an isotype of scFv	[160]
T-lymphocytes						
	CD3 T-cell receptor	Anti-CD3 antibodies	PLL polyplexes	pDNA	1000-fold enhanced gene expression compared to unmodified PLL and Tf-PLL in T-cells; successful transfection to primary human lymphocytes	[58]
	CD4	Anti-CD4 mAb	LNP	siRNA	Specific delivery to CD4+ cells ex vivo; gene silencing activity was observed in blood, bone marrow, spleen and lymph nodes	[59]
	Ly6c	Anti-Ly6-mAb	LNP	mmRNA	Targeted delivery to Ly6c positive cells *in vitro*; *in vivo* evaluation in IBD mouse model showed increased protein expression [20-fold in intestine, tenfold in spleen]; expression of anti-inflammatory IL-10 after delivery of IL-10 encoding mmRNA	[164, 165]
	n.a	None (chemical targeting)	LNP	Barcode siRNA, sgRNA	Screening of 168 different LNP formulations *in vivo* with variations of head group, lipid alkyl chains, phospholipid and molar composition; adamantyl-DSPC delivered siRNA and sgRNA to T cells (and Kupffer cells)	[166]
	n.a	None (chemical targeting)	LNP	siRNA	Variation of head group and alkyl chain; piperazine headgroup led to accumulation and gene silencing in the spleen	[167]
	Integrin β_7_	Anti-β_7_-mAb	LNP	siRNA	CD45 mRNA silencing in CD4+ and CD8+ T cells in spleen and lymph nodes	[167]

Abbreviations: PLL, Poly-L-lysine; Man, Mannose; pAsp(DET) poly(N-[N-[2-aminoethyl]-2-aminoethyl] aspartamide); CM Mannose, chemically modified mannose; PEI, polyethylene imine; BSA, bovine serum albumin; DCs, dendritic cells; scFv, single chain antibody; CD, cluster of differentiation; Tf, Transferrin; mAb, monoclonal antibody; Ly6c, lymphocyte antigen 6 complex; mmRNA, modified messenger RNA; IBD, inflammatory bowel disease; mAb, monoclonal antibody

DNA delivery to DCs *via* mannose receptor was described by Diebold *et al*. by using mannose functionalized PEI polyplexes. Receptor-specific uptake was demonstrated in a competition assay with mannose albumin, which lowered gene expression of Man-polyplexes [82]. Gao *et al*. designed mannose ligands optimized towards the carbohydrate recognition domains of mannose receptor and the DC-specific intercellular adhesion molecule-3-grabbing non-integrin (DC-SIGN, CD209), respectively. Liposome uptake by DC2.4 cells and DC-SIGN expressing HEK293 cells was observed in a ligand-dependent manner. These particles were not yet used to deliver nucleic acids to DCs but provided deeper insight in the understanding of ligand design to improve receptor interaction [155]. The impact of PEG spacer lengths on particle size, stability and transfection efficacy was also examined using Man-PEG-cholesterol lipids in mRNA-LNP formulations. It could be shown that zetapotential and particle size remained unchanged by increasing PEG lengths, whereas PEG1000 showed highest transfection efficacy while maintaining serum stability [156]. Recently, the effect of mannan-coating of LNPs for the delivery of RNA vaccines was investigated by using mono- and multivalent mannose residues linked to cholesterol, resulting in an increased immunization arguing for the use of targeted, mannan-functionalized RNA vaccines [157].

Targeting of dendritic cells was also achieved *via* DEC205, another receptor from the mannose receptor family [158, 159]. Katakowski *et al*. formulated LNPs bearing a single-chain antibody to target DEC205 expressing murine DCs. Receptor-specific binding and uptake of the siRNA-LNPs were determined *via* flowcytometry, showing that uptake was twofold improved by targeted LNPs. DEC205-dependent internalization was further confirmed by reduced knock-down efficacy in DEC205 deficient mice [160].

The spleen and more specifically DCs were chemically targeted by lipoplexes with an inversed lipid/RNA charge ratio giving negatively charged particles. After systemic administration, these particles successfully delivered mRNA encoding for antigens and promoted stimulation of APCs for cancer immunotherapy [161].

### T-lymphocytes

T-lymphocytes also play a crucial role in cellular immune response [162]. Therapy of immune related disorders, e.g., inflammation or cancer, can be achieved by RNAi in T-lymphocytes. As gene delivery in these cells has appeared to be challenging [147, 163], the carrier systems have been decorated with ligands, mostly monoclonal antibodies, to improve nucleic acid delivery. For example, Buschle *et al*. achieved gene transfer to human T-lymphocytes by decorating Tf-PLL polyplexes with antibodies against the CD3 T cell receptor [58]. Ramishetti *et al*. aspired to deliver siRNA-LNPs to CD4 + T-lymphocytes by attachment of anti-CD4-monoclonal antibody, whereby the specific delivery to CD4 + lymphocytes could be confirmed ex vivo. Additionally, i.v. injection in mice showed gene silencing activity in spleen, lymph nodes, bone marrow and blood [59]. Veiga *et al*. evaluated LNPs loaded with modified mRNA for delivery to Ly6c + inflammatory leukocytes [164]. For this purpose, the targeting ligand was attached to the LNP *via* incorporation of lipoproteins interacting with antibodies [165]. Decoration with targeting mAbs towards inflammatory leukocytes led to strongly increased interleukin-10 expression in spleen and intestine [164].

Great advances regarding nucleic acid delivery to T-lymphocytes were made using the chemical targeting approach. Using the barcode screening technology, Lokugamage *et al*. studied the distribution of a library of 168 siRNA-LNP formulations with structural changes regarding the lipids in different cell types *in vivo* and showed that constrained LNPs preferably delivered the genetic material to splenic T lymphocytes instead of hepatocytes making these formulations interesting for immunotherapy [166].

A screening of 14 structurally different ionizable lipids by variation of linker backbone, head group and alkyl chains for delivery of siRNA-LNPs into leukocytes was reported by Ramishetti *et al*. The biodistribution after i.v. injection in mice showed accumulation in spleen for piperazine head group and in liver for tertiary amine head group [167]. In the same study, specificity was further improved by combination of both, chemical and active targeting. Decoration of the LNPs with anti-integrin β-mAbs resulted in CD45 mRNA knockdown in CD4 + and CD8 + lymphocytes in spleen and lymph nodes. Nevertheless, only a limited gene silencing ability was detected overall [167].

## Lung as Target

Many severe, eventually lethal diseases are associated with the lung, for example cystic fibrosis, chronic obstructive pulmonary disease (COPD), asthma or pulmonary fibrosis amongst others. One advantage of nucleic acid delivery to the lung is certainly the accessibility of the lung *via* local and systemic administration routes. However, protective mechanisms and physiological barriers such as mucosal barrier or immune cells may impair the delivery of nucleic acids [168]. For selective, targeted nucleic acid transfer into the lung chemical as well as receptor-mediated targeting strategies have been evaluated with the key results summarized in **Table ****IV**.

**Table IV:** Receptor-Mediated Non-Viral Nucleic Acid Delivery to the Lung

Receptor	Ligand	Delivery System	Type of nucleic acid	Key results	Reference
Insulin receptor	Insulin	PEI polyplex	pDNA	Selective delivery to alveolar epithelial cells	[91]
Lactoferrin receptor	Lactoferrin	PEI polyplex	pDNA	Selective delivery to bronchial epithelial cells	[81]
Integrin	RGD motif	Liposome	pDNA	High transfection efficacy in lung endothelial cells for targeted liposomes *in vivo*	[54]
	TAT-RGD motif	Direct conjugate	pDNA	Enhanced uptake of targeted particles by pulmonary cells	[176]
		Cationic liposome	pDNA	Fivefold increased gene expression in A549 cells compared to lipofectamine	
	RGD motif	Lipoplex	pDNA	Significantly improved transfection efficacy for RGD-bearing polyplexes	[177]
Polymeric IgR	Antisecretory component antibody	PLL polyplex	pDNA	Proof of concept for transfection efficacy to human tracheal epithelial cells, competition assay blocked uptake of targeted polyplexes	[178]
PECAM	Anti-PECAM antibody	PEI polyplex	pDNA	Enhanced gene transfer efficacy and reduced toxicity	[179]
	Anti-PECAM antibody	LNP	mRNA	Enhanced protein expression in lung endothelial cells; reduced accumulation in hepatocytes	[84]
Transferrin receptor	Transferrin	PEI polyplex	siRNA	Enhanced uptake of Tf-PEI polyplexes by pulmonary ATCs *in vitro* and in asthma mouse model after intratracheal application; improved endosomal escape by addition of melittin, 40% more effective than lipofectamine	[180, 181]
β2-adrenoceptor	Clenbuterol	PEI polyplex	pDNA	Enhanced gene expression in alveolar epithelial cells	[50]
	Salbutamol	Chitosan polyplex	siRNA	Delivery to bronchial epithelial cells	[51]
IP1	Iloprost and Treprostinil	PEI polyplex	pDNA	Enhanced transfection efficacy	[52]
Lectins	Galactose Glucose Lactose	PLL polyplex	pDNA	Improved, sugar-type dependent gene expression in cystic fibrosis airway epithelial cells	[185-188]
	Galactose	PEGylated PEI polyplex	pDNA	Increased transfection efficacy *in vitro* and *in vivo*	[189]

Abbreviations: PEI, polyethylene imine; RGD, arginine-glycine-aspartic acid; TAT, transactivated transcription peptide; Ig, immunoglobulin receptor; PLL, poly-L-lysine; PECAM, platelet endothelial cell adhesion molecule; IP1, prostacyclin receptor; PEI, polyethylene imine

Due to their positive surface charge, many polyplex (PEI) as well as cationic liposome formulations automatically accumulate in the lung when injected systemically [169–172]. In addition, efforts were made to generate lung targeted LNPs, which typically accumulate in hepatocytes, by modification of lipid composition and the type of ionizable lipid. In the course of the development of “SORT”-LNPs, it was reported that increasing amounts of the positively charged component DOTAP shifted accumulation from hepatocytes to lung endothelial cells [108]. Chemical targeted synthetic carriers for specific lung delivery of mRNA and pDNA were developed by Kaczmarek *et al*. The hybrid polymer-lipid formulations used in their studies, consisting of poly(β-amino esters) (PBAEs) and PEGylated lipids, generated protein expression in the lung after i.v. injection in mice, but not in other organs [173–175].

Active targeting of lung epithelial cells resulting in receptor-mediated uptake of non-viral delivery systems was obtained by several classes of ligands, such as peptides, proteins, antibodies, carbohydrates and also small drugs. Elfinger *et al*. studied the selective delivery to different lung epithelial cell types. It was reported that pDNA/PEI polyplexes modified with lactoferrin delivered the nucleic acid selectively to bronchial epithelial cells *via* lactoferrin receptor, but not alveolar epithelial cells, whereas adsorption of insulin to pDNA/PEI polyplexes showed increased luciferase gene expression in alveolar epithelial cells, but not in bronchial epithelial cells [81, 91]. Integrins are also abundantly found on lung cells and attempts were made to achieve receptor-mediated uptake *via* caveolae-dependent pathway by incorporation of arginine-glycine-aspartic acid (RGD) motifs into the delivery system [54, 176, 177]. More specific targeting of the lung was achieved using antibodies as ligands. In early studies, Ferkol *et al*. observed targeted delivery of pDNA-polylysine complexes conjugated with Fab fragments of immunoglobulins directed against the polymeric immunoglobulin receptor (IgR) which is involved in the transport of immunoglobulins A and M from cell surface into lung epithelial cells. A competition assay with excess of Fab ligand blocked delivery gives further evidence for receptor-mediated uptake [178]. Additionally, lung-specific nucleic acid transfer was accomplished using antibodies directed against the platelet endothelial cell adhesion molecule (PECAM). For example, Li *et al*. were able to generate higher gene expression after i.v. injection of anti-PECAM-mAb decorated pDNA-PEI polyplexes in mice, furthermore observing reduced cytotoxicity when using ligand-modified carriers [179]. More recently, mRNA-LNPs, which are known to accumulate in the liver, were modified with monoclonal antibodies directed against PECAM-1. Intravenous injection in mice avoided accumulation in hepatocytes but resulted in enhanced protein expression in lung endothelial cells [84].

Transferrin (Tf) was used as targeting ligand to mediate the delivery of PEI-siRNA polyplexes to pulmonary activated T cells (ATCs). A study by Xie *et al*. has shown that transferrin modification led to enhanced cellular uptake and efficient, selective gene knockdown *in vitro* as well as in an asthma mouse model after intratracheal application [180]. Further optimization of the Tf-PEI polyplexes by blending with PEI bearing the endosomolytic peptide melittin improved endosomal escape capability of the cargo resulting in enhanced cellular uptake [181]. In fact, optimization of endosomal escape properties for delivery of siRNA polyplexes to the lung has been subject of further studies. For example, Pun *et al*. developed a virus-inspired polymer for endosomal release (VIPER) [182], which was applied for efficient pulmonary delivery of siRNA both *in vitro* and *in vivo* [183]. VIPER/siRNA polyplexes also showed antiviral effect by promoting suppression of viral replication of SARS-CoV-2 *ex vivo* in human lung tissues and in mouse models [184].

Furthermore, small chemical compounds, which have already been used effectively as drugs for asthma treatment were used as targeting ligands coupled to synthetic nucleic acid carriers for targeted nucleic acid delivery to the lung. For example, agonists for the β2-adrenoceptor were successfully used for targeted and improved delivery of nucleic acids to lung epithelial cells. Elfinger *et al*. demonstrated enhanced gene expression in alveolar epithelial cells *in vitro* as well as *in vivo* after inhalation of Clenbuterol-functionalized polyplexes [50]. Specific delivery of siRNA to bronchial epithelial cells could be improved by coupling of Salbutamol to the formulation, as shown by Luo *et al*. using guanidinylated chitosan carriers [51]. In addition, PEI-polyplexes modified with Iloprost and Treprostinil, prostacyclin derivatives targeting the prostacyclin receptor IP1, also exhibited enhanced transfection efficacy of pDNA polyplexes in lung epithelial cells as well *in vivo* in the lungs of mice after aerosol administration [52]. For these chemical ligands, their possible dual role as drugs was not explored.

Additionally, lectins have been studied for lung-specific uptake of non-viral delivery systems. Several studies by Kollen *et al*. showed that gene expression of pDNA/polylysine polyplexes could be increased through functionalization with β-galactose, α-glucose as well as lactose compared to other monosaccharides and the non-targeted formulation after transfection to cystic fibrosis cells [185–188]. Transfection of galactosylated polyplexes also resulted in improved gene expression compared to non-targeted polyplexes in A549 cells as well as *in vivo* experiments after intratracheal administration [189]. In both studies, lectins were hypothesized to play a role in the specific uptake of the particles, although the particular uptake route was not further addressed.

## Brain as Target

Many neurodegenerative disorders such as Alzheimer’s disease, Huntington’s disease, Parkinson’s disease or amyotrophic lateral sclerosis (ALS) originate in the central nervous system (CNS). Treatment of these diseases *via* systemic administration routes remains challenging due to poor accessibility of the brain through the blood–brain barrier (BBB). Therapeutic nucleic acids compacted into synthetic carrier systems are not able to cross the BBB *via* diffusion [190]. However, nucleic acid delivery to the brain *via* systemic administration is highly desired, as topic routes like intracranial or intracerebroventricular injections as well as physical methods that enhance the permeability of the BBB are highly invasive. Thus, synthetic carriers must be decorated with ligands, which are recognized by receptors or carriers embedded in the BBB, becoming “trojan horses”, which are enabled to deliver nucleic acid to the brain through receptor-mediated transcytosis (RMT) or carrier-mediated transcytosis (CMT). Various synthetic carrier systems were modified with several ligands, ranging from proteins, peptides and aptamers to generate brain-targeted gene delivery (see Table V).

**Table V:** Receptors and Ligands for Targeted Delivery of Nucleic Acids to the Brain

Receptor	Ligand	Delivery system	Type of nucleic acid	Key findings	Reference
Transferrin receptor	Transferrin	Lipoplex	siRNA	Efficient gene silencing in primary murine cortical neuronal cells and *in vivo* without cytotoxicity	[193]
	Transferrin	PEG-PAMAM dendrimer polyplex	pDNA	Enhanced gene expression in BCECs and in mice brain	[194]
	Transferrin	PPI polyplex	pDNA	Targeted polyplexes accumulated in mice brain	[195]
	Anti-TfR-mAb	Immunoliposome	pDNA	TfR-mAb promoted both, crossing of BBB and delivery to TfR-expressing glioma cells	[196]
	re-TfR-peptide	Lipo-oligo(amidoamine) polyplex	pDNA	Enhanced luciferase gene expression in N2a cells compared to non-targeted lipoplexes and scrambled peptide ligand	[55]
Lactoferrin receptor	Lactoferrin	PEG-PAMAM polyplex	pDNA	2.2-fold increased gene expression *in vivo,* selective gene transfer to the brain	[199]
	Lactoferrin	PPI Polyplex	pDNA	2.1-fold increased gene expression *in vitro*, significantly higher gene expression *in vivo*	[200]
LRP1	Angiopep-2	PEG-PAMAM dendrimer polyplex	pDNA	Selective uptake of polyplexes by BCECs, accumulation of targeted polyplexes in brain, untargeted in spleen	[205]
	Angiopep-2	LNP	siRNA	*In vitro* study of cellular uptake and gene silencing efficacy in U87MG and b.End3 cells	[206]
nAChR	RVG29	Oligoarginine polyplex	siRNA	Enhanced gene expression in brain after i.v. injection, but not in other organs	[212]
	RVG29	PEG-PAMAM dendrimer polyplex	pDNA	Brain accumulation after systemic administration, GABA receptor involved in uptake	[213]
	RVG29	PEI polyplex	miRNA	Reduced signal of reporter gene due to silencing activity, accumulation in brain	[267]
	RVG29	Trimethylated chitosan polyplex	siRNA	Efficient gene silencing of BACE1, accumulation in brain	[214]
	RVG29	Poly(mannitol-co-) PEI polyplex	siRNA	Efficient gene silencing of BACE1	[215]
	RVG29	Exosomes	siRNA	Gene knock-down of BACE1	[216]
	RVG-9r	SNALP	siRNA	Efficient silencing of Machado Joseph disease involved proteins *in vitro* and *in vivo*	[217]
Laminin receptor	EPRNEEK	Dendrigraft PLL polyplex	DNA	Improved uptake and gene expression by exogenous ligand compared to endogenous laminin ligand	[218]
Leptin receptor	Leptin-30 peptide	PEGylated PLL Polyplex	pDNA	Improved transfection efficacy in BV-2 cells; accumulation in brain after i.v. injection	[210]
VCAM1	Anti-VCAM-antibody	LNP	mRNA	Specific mRNA delivery to inflammatory brain, but not to leukocytes, expression of anti-inflammatory protein	[219]
GLUT1	Glucose	Polyplex	ASO	Accumulation in mice brain after i.v. injection depending on glucose-concentration	[223]

Abbreviations: PAMAM, poly(amidoamine); BCECs, brain capillary endothelial cells; PPI, polypropylene imine; TfR-mAb, transferrin receptor monoclonal antibody; re-TfR, retro-enantio transferrin receptor, N2a, neuro2a cell line; LRP-1, low-density lipoprotein receptor related protein 1; GLUT1, glucose transporter 1; nAChR, nicotinic acetylcholine receptor; RVG29, rabies virus derived 29-mer peptide; GABA, gamma-aminobutyric acid; BACE1, beta-secretase 1; SNALP, stable nucleic acid lipid particle; PLL, poly-L-lysine; VCAM1, vascular cell adhesion molecule 1

A possible approach to facilitate nucleic acid transfer into the CNS is to use receptor-mediated transcytosis by transport proteins that enable the passage of essential nutrients, proteins or lipids across the BBB.

### Receptor-Mediated Transcytosis

#### Receptors for Iron Transport Proteins

Transport of iron across the BBB is mediated by several iron transport protein receptors, including transferrin, lactoferrin or melanotransferrin. Above all, transferrin receptor was widely studied for targeted uptake of synthetic carriers to the CNS. As known from previous studies, the transferrin protein itself holds great potential to mediate receptor-dependent polyplex uptake by TfR-expressing cells and therefore, was explored for mediating transcytosis across the BBB [191, 192]. For example, Cardoso *et al*. prepared Tf-modified siRNA-lipoplexes that showed superior uptake by neuronal cells as well as significant gene silencing in both, *in vitro* and *in vivo* compared to non-targeted particles [193]. Moreover, dendrimer-polyplexes based on PAMAM or PPI were functionalized with human transferrin, resulting in successful pDNA delivery across the BBB. Biodistribution studies further confirmed accumulation of TfR-targeted dendrimers in the brain [194, 195]. TfR was also targeted by immunoliposomes bearing monoclonal antibodies (mAb) directed against rat TfR for RNAi therapy *via* delivery of plasmids encoding for short hairpin RNA (shRNA). It could be demonstrated that TfR-targeted immunoliposomes provided a dual targeting effect, as they promoted BBB crossing and subsequent uptake of glioma cells, which are also overexpressing TfR [196]. Recently, TfR-mediated delivery to neuronal cells was successfully accomplished by our group using a retro-enantio peptide sequence that showed high affinity towards the transferrin receptor. The “retro-enantio” approach provides stability against peptide degradation by inversion of the peptide order and usage of D-configurated amino acids while maintaining receptor binding affinity [197]. The retro-enantio ligand was conjugated to sequence defined lipo-oligo(amidoamines) (lipo-OAAs) for the delivery of both, siRNA and pDNA, to N2a cells [55].

#### Lactoferrin Receptor

Besides TfR, the lactoferrin receptor (LfR) is also involved in cellular iron uptake and expressed on the BBB [198]. By using LfR, increased gene expression of dendrimer polyplex formulations bearing lactoferrin (Lf) could be observed. Lf-modification of PAMAM dendrimer polyplexes resulted in 2.2-fold increase of luciferase gene expression *in vivo* compared to untargeted particles. In addition, selective delivery to the brain was reported after systemic administration [199]. Additionally, lactoferrin-PPI-dendrimers showed improved transfection in b.End.3 cells and significantly increased accumulation in mice brain [200].

#### Melanotransferrin

Furthermore, a short 12-amino acid peptide which has shown interaction with the iron transport protein melanotransferrin was able to mediate entry in the brain. Conjugation to siRNA enabled not only accumulation in the brain, but also *in vivo* gene silencing of NOX4, a gene that is upregulated during stroke [201, 202].

#### LRP1

Nucleic acid transfer across the BBB was furthermore achieved by low-density lipoprotein receptor related protein (LRP1) mediated transcytosis. Demeule *et al*. developed Kunitz domain derived peptides from aprotinin, named Angiopep, that showed the ability to overcome BBB *via* LRP1-mediated transport [203, 204]. Angiopep-2 was attached to DNA/PAMAM-dendrimers showing selective uptake by BCECs *in vitro* and a shift of the biodistribution from spleen (for untargeted carriers) to brain (for targeted polyplexes) *in vivo* [205]*.* The same ligand was later included in siRNA-LNP formulations by Bruun *et al*. for *in vitro studies* on uptake and gene silencing activity in human glioblastoma U87MG and murine brain endothelial bEnd.3 cell line. In addition to an increased gene knock-down, it could be observed that uptake could be improved about 2.4-fold by Angiopep-2 modification [206]. Moreover, a novel artificial ligand named L57 was found to enable BBB crossing *in vivo* by interaction with LRP1 [207]. Compared to Angiopep-7, L57 showed enhanced CNS uptake capability and low cytotoxicity [208].

#### Leptin Receptor

Moreover, the leptin receptor, which is responsible for recognition and transcytosis of the appetite regulating peptide leptin, was used for nucleic acid delivery to the brain [209]. It was shown that a leptin-derived 30-amino acid peptide attached to poly-L-lysine carrier was able to generate improved DNA transfection in BV-2 cells and accumulation in mice brain after i.v. injection [210].

#### Pathogen-Derived Peptide Ligands

In addition to ligands interacting with receptors which transport essential molecules across the BBB, another approach is to modify the carrier system with peptides deriving from viruses, bacteria or venoms that naturally show capabilities to enter the brain.

For example, the peptide RVG29 was studied for nucleic acid transfer across the BBB. The ligand derived from rabies virus glycoprotein (RVG), which naturally shows the ability to enter the brain as part of its pathology and targets mainly the nicotinic acetylcholine receptor (nAChR) [211]. RVG29 was included in polyplex and LNP formulations yielding nucleic acid transfer across the BBB. For example, Kumar *et al*. [212] reported efficient gene silencing using RVG-bearing siRNA oligo(arginine) formulations. A study by Liu *et al*., using RVG29-PEG-PAMAM/DNA polyplexes, revealed involvement of GABA receptor in uptake [213]. Efficient gene silencing of BACE1, a protein involved in Alzheimer’s disease, could be achieved by several groups using polyplex as well as exosome formulations that were functionalized with RVG29 [214-216]. Furthermore, a lipid-containing formulation bearing RVG-oligo(arginine) residues exhibited the ability to silence a mutant ataxin-3, involved in the pathology of Machado-Joseph disease, a hereditary ataxia disorder [217].

Furthermore, Liu *et al*. demonstrated the capability of a peptide sequence derived from meningitis-causing pathogen to mediate brain-specific delivery of surface-modified PLL/DNA-dendrimers via laminin receptor, resulting in enhanced cellular uptake by BCECs and U87MG cells compared to an endogenous laminin-targeting ligand [218].

#### VCAM1

A recent study by Marcos-Contreras *et al*. explored the specific delivery of antibody-modified mRNA-LNPs using an anti-vascular cell adhesion molecule 1 (anti-VCAM1) antibody to the inflammatory brain. It was found that the particles were delivered to brain endothelial cells but not to leukocytes. Furthermore, as a consequence of successful mRNA delivery, expression of anti-inflammatory thrombomodulin could be observed in a mouse model [219].

#### Direct Conjugates for BBB-Targeting

Some of the aforementioned ligands, such as Angiopep and RVG-29, as well as further peptide ligands were used for direct conjugation to phosphorodiamidate morpholino oligomers (PMOs), which function as splice-switching oligonucleotides. It was demonstrated that a truncated peptide-derivative of ApoE mediates PMO delivery in the CNS [220].

### Carrier-Mediated Transcytosis

#### Transcytosis *via* GLUT1

An additional pathway to overcome BBB is through transcytosis by glucose transporter 1 (GLUT1). As transport carrier for glucose, GLUT1 is abundantly expressed on brain capillary endothelial cell (BCEC) membrane, ensuring adequate glucose supply of the brain [221]. Researchers exploited this transport mechanism by using glucose modified nanoparticles as “trojan horses” to induce gene transfer into the brain and other GLUT1-rich cells. For example, Kataoka *et al*. developed glucose-decorated polymeric carriers, which facilitated nanoparticle delivery to GLUT1-rich cancer cells under glycemic control [62, 222]. These block-copolymers were applied for the delivery of ASOs to the brain via intravenous injection into mice, providing efficient gene knockdown [223]. Another glucose-ligand capable of crossing the BBB is an opioid-derived glyco-heptapeptide (g7). Even though the transport mechanism of g7-functionalized particles into the brain is not fully understood, decoration of nanoparticles with g7 showed brain specific uptake after i.v. injection into rat and therefore presents a promising ligand for nucleic acid delivery to the CNS [224-227].

## Ocular Targeting

### Retina

Many genetic eye disorders, either inherited or environment-dependent, may lead to loss of vision eventually due to impaired functions of photoreceptors or retinal pigment endothelium (RPE). Therapeutic approaches mostly use classic gene therapy with DNA for gene replacement or gene-editing methods. Formulations are usually injected subretinal or intravitreal due to the blood ocular barrier and in order to reduce off-target effects or elimination by immune system. Furthermore, persistent high levels of gene expression after single injections are highly desired for retinal gene therapy. Sustainable gene expression was achieved by using PEG-PLL/DNA polyplexes, which were locally injected in mouse models [228-230]. Delivery of nucleic acid therapeutics to the retina resulting in long-term gene expression was also reported by the group of Zheng-Rong Lu [231]. Lu and co-workers have developed a multifunctional ionizable lipid, called “ECO”, which served as efficient gene carrier system in several applications [232-234]. A hybrid ECO/G4-dendrimer formulation was applied successfully as carrier system for GFP reporter gene to human ARPE-19 cells and in animal experiments [235]. Moreover, ECO served as carrier for the therapeutic ABCA4 plasmid, supported by a rhodopsin promoter, for the treatment of Stargardt disease. These formulations provided up to 8 months of gene expression and disease progression delay for 6 months in ABCA4 deficient mice [236]. ECO/DNA formulations were also functionalized with PEG_3.4 kDa_-all-*trans*-retinylamine to target the interphotoreceptor retinoid binding protein (IRBP), a key protein in the retinoid cycle [237]. In this study, high transfection efficacy of the reporter gene GFP could be demonstrated in ARPE-19 cells and mouse models of Leber’s congenital amaurosis (LCA) type 2 after subretinal injection with high expression levels up to 120 days [238]. Additionally, a chemically stable retinoid analogue ACU4429, linked to the carrier system via pH-sensitive hydrazone-PEG_3.4 kDa_-spacer, was used for IRBP-mediated delivery of ECO/DNA particles and gene expression of ABCA4 in ARPE-19 cells as well as in *abca4*^*-/-*^ mice [239].

Furthermore, liposome-protamine complexes have shown potential to promote long term gene expression or gene knock-down, respectively [240-242]. More recently, LNP formulations encapsulating either mRNA [243] or siRNA [244] were screened to investigate cell-specific retinal nucleic acid transfer depending on surface charge and LNP composition. It was observed that mRNA-LNPs containing ionizable lipid are preferably internalized by RPE cells, suggesting ApoE-mediated uptake, whereas formulations with permanently cationic lipids showed only low transfection efficacies in the retina [243]. Another study using siRNA-LNPs demonstrated that LNPs with positive zetapotential around +35 mV distributed preferably in the vitreous and retina after local injection [244].

### Cornea

In order to address corneal gene delivery, researchers used hyaluronic acid as a targeting ligand for the CD44-receptor, which is expressed by human corneal epithelial cells and is responsible for turnover of HA [245-247]. For instance, de la Fuente *et al*. developed HA-chitosan nanoparticles loaded with reporter DNA which successfully transfected human corneal epithelial (HCE) cells and conjunctiva cells via the CD44-receptor [248, 249]. Further investigations of the internalization pathway revealed that the particles were endocytosed by caveolae-mediated endocytosis [49, 250]. CD44-receptor mediated intracorneal uptake was also assessed by Hornof *et al*. using HA-coated PEI/DNA polyplexes demonstrating that nanoparticles coated with low-molecular weight HA generated well shielded, stable particles while maintaining efficient transfection activity [48].

## Muscle as Target

The delivery of nucleic acids to skeletal and cardiac muscles allows the treatment of muscle-related disorders such as muscular dystrophy. Efforts were made to develop lipid-siRNA conjugates that enable muscle-targeted delivery upon systemic injection. Therefore, a library of lipid-ASO conjugates were screened regarding their ability to deliver functional ASOs to muscle cells after i.v. injection in mice. It was demonstrated that delivery to muscle cells was dependent on the length of the fatty acid, with C16 to C22 showing highest accumulation based on their affinity to albumin which mediated transport to muscle cells [251]. The palmitic-ASO conjugate was further evaluated revealing a slight increase in ASO activity, but relatively rapid clearance [252]. A following study evaluated ASO potency and association with human, rodent and monkey plasma proteins, showing a preferred binding of palmitate-ASOs to human and rodent albumin as well as histidine-rich glycoprotein possibly explaining enhanced ASO activity in the muscle. Additionally, an enhanced *in vivo* ASO potency was observed in rodents, but only a modest improvement in monkeys [253]. Moreover, different lipids were conjugated to chemically stabilized siRNA for a distribution study in mice. Most formulations accumulated in clearance organs such as liver or kidney, while docosanoic acid-siRNA conjugate (DCA) delivered siRNA partly to other tissues [254]. Compared to cholesterol-siRNA, delivery of DCA-conjugates to skeletal and cardiac muscles was enhanced about threefold and 2.5-fold, respectively. Using DCA conjugates, sustained silencing of myostatin mRNA in muscles was obtained leading to reduced myostatin protein levels and promotion of muscle growth after systemic injection [255].

## Active targeting: *In Vitro Versus In Vivo*

It is noticeable that delivery systems using targeting ligands are rather rare on the medical market. Only a few products such as GalNAc direct siRNA conjugates and ApoE endogenously targeted Patisiran were approved by FDA. In fact, the majority of targeting ligands was evaluated in cell culture studies, demonstrating improved *in vitro* performance. However, most formulations have not taken the step to product development for *in vivo* applications.

Potential reasons for this observation must be considered and evaluated in order to find explanations for this translational bottleneck. There is a great discrepancy between the results obtained from *in vitro* studies and *in vivo* performance, making predictions for (pre-)clinical studies questionable when drawn from cell culture evaluation [38-40].

**Fig. 5:**
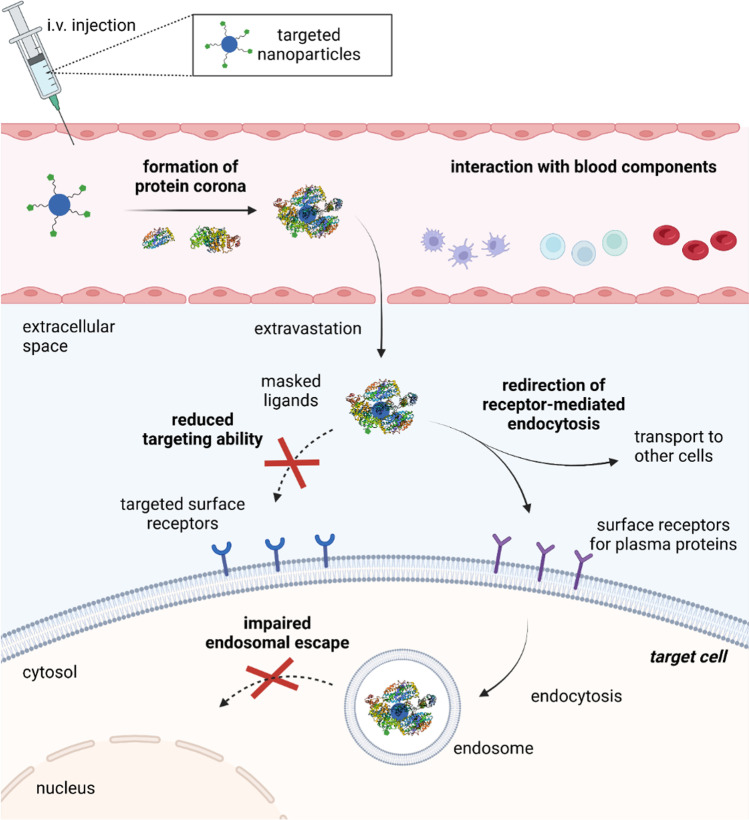
Interaction of i.v. injected targeted nanoparticles with blood components and consequences for the delivery process. Formation of protein corona leads to reduced intended targeting ability due to masked ligands. Protein corona may lead to transport to other cells or uptake via receptors recognizing plasma proteins. Additionally, endosomal escape can be hampered by protein layer. Created with BioRender.com

*In vivo* nucleic acid delivery appears to be affected by several barriers. In contrast to small molecule drugs, nucleic acids exhibit unfavored pharmacokinetic and pharmacodynamic profiles due to their high molecular weight and charge, excluding membrane diffusion as internalization route. The demand for endocytic internalization pathways, membrane barriers become more difficult to overcome and formulation properties have to be carefully tailored, resulting in complex, multi-component nanoparticle formulations. Furthermore, different types of nucleic acid therapeutics have different requirements on the formulation. Therefore, addressing new obstacles, i.e., cellular barriers such as nanoparticle uptake and endosomal escape represent major bottlenecks for clinical translation, as discussed in detail by several researchers [256-259]. Prior to cellular uptake and endosomal escape, interaction of carriers with blood components determine not only transport but eventually also efficiency. Thus, the behavior in plasma represents a critical obstacle for the delivery system. Depending on their physicochemical properties, including size, zeta potential and surface modification, certain proteins will adsorb on their surface to form a “protein corona”. This protein corona largely determines the characteristics of the particles in the organism, i.e., biodistribution, pharmacokinetics and immunogenicity. It was observed that the adsorption of plasma proteins leads to reduced accessibility or interaction between exogenously incorporated ligands and their targeted receptors (Fig. 5). For example, several studies reported that transferrin-coated nanoparticles showed reduced or lacking specificity towards TfR in presence of the protein corona as a result of ligand blockade [260-262]. The resulting “biological identity” was highly dependent on the composition of the protein corona. In particular, *in vitro* protein corona depleted targeting capability, whereas a protein corona resembling *in vivo* conditions caused only a reduction in specific receptor-mediated uptake [261].

Interestingly, two studies could prove that the *in vivo* protein corona even enhanced the overall uptake of the nanoparticles, hypothesizing redirection of cellular uptake by the protein layer and opening new paths for particle internalization [262, 263]. As a consequence, plasma protein adsorption could also lead to redirected transport to off-target cells such as the RES, which hampers selective nucleic acid delivery and efficacy. In addition, Tonigold *et al*. observed that antibodies covalently bound to nanoparticles lose their targeting ability almost completely in presence of serum. Particles with physically adsorbed antibodies, however, maintained their targeting ability, probably due to the fact that these antibodies were not completely exchanged or masked by serum proteins [85].

**Fig. 6:**
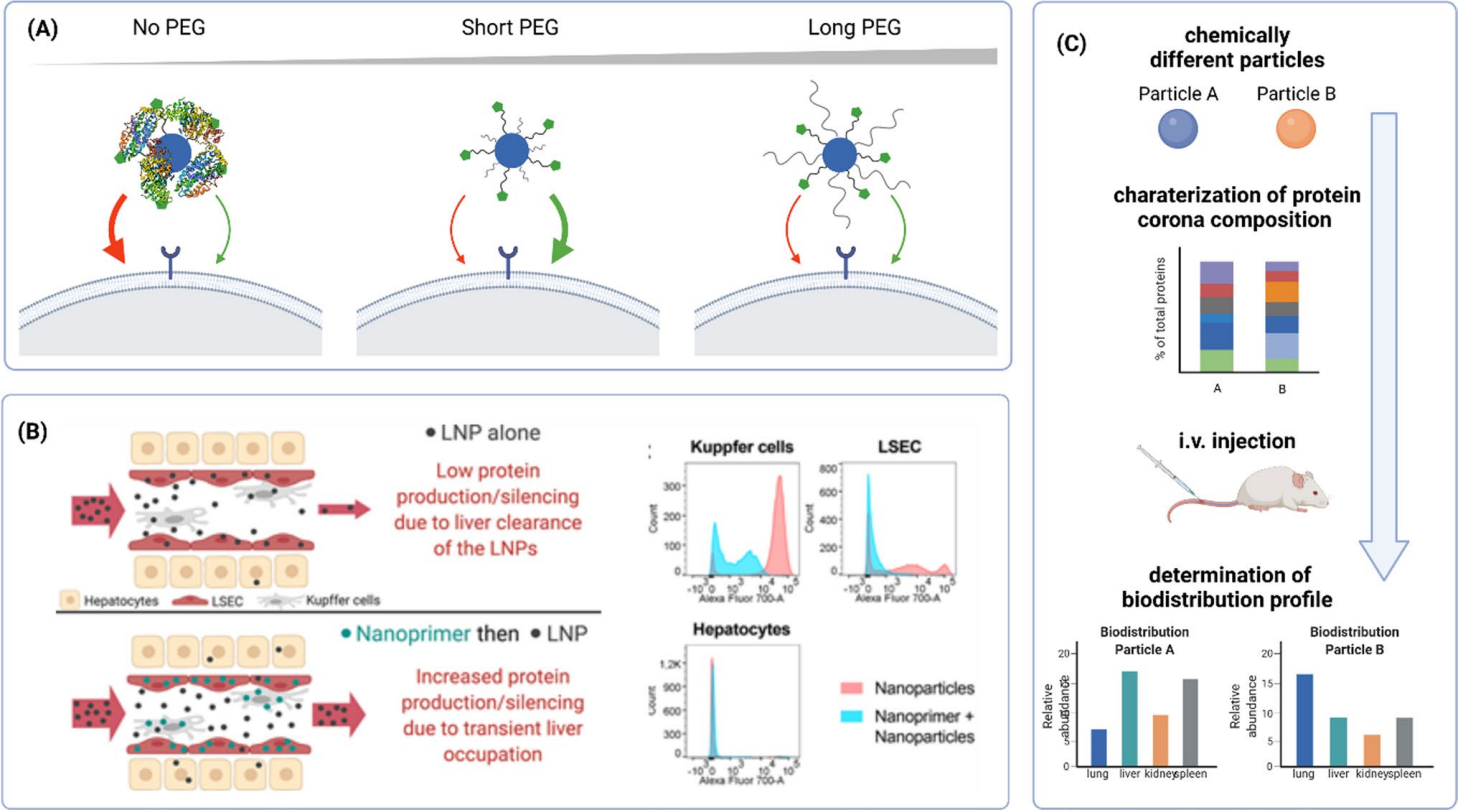
Strategies to improve or maintain targeting ability *in vivo*. (**A**) PEG-Backfilling avoids formation of protein corona, which would mask targeting ligands. Short PEG chains are necessary to maintain accessibility of the ligands [264]. (**B**) Application of a nanoprimer is reducing off-target LNP uptake by Kupffer cells and LSECs and enhances delivery to hepatocytes. [266]. Reproduced with permission from reference with Copyright © 2020, American Chemical Society. (**C**) Adjustment of surface chemistry leads to modified protein corona composition and can be used for targeted delivery by altered biodistribution profile. Created with BioRender.com

Traditionally, masking the surface properties causing plasma protein adsorption is a key strategy to overcome this issue and to maintain targeting ability. PEGylation has proven to reduce undesired interactions and consequently retain ligand-accessibility which is essential for targeted delivery *in vivo* [41]. One approach to further attenuate particle interaction with plasma proteins is PEG “backfilling” (Fig. 6A). Therefore, the surface of gold nanoparticles (Au-NPs), functionalized with targeting transferrin-PEG(5 kDa) was further modified at free reaction sites with PEG spacers of varying lengths, preventing the particles from protein corona formation. In order to enable receptor-recognition, PEG chains for shielding had to be shorter than the spacer between particle and targeting ligand [264]. Despite these difficulties, there are promising examples which have already demonstrated efficient, cell- or tissue-specific delivery of nucleic acids. Additionally, scientists have started to exploit the protein corona to tune particle distribution *in vivo*. It is commonly known that plasma proteins adsorption depends on particle surface characteristics. Hence, slight structural changes of the particle surface have shown to affect the protein corona composition. As a consequence of protein corona modification, transport to target cells by coating with certain plasma proteins which serve as endogenous ligands can be obtained *in vivo*. For example, DOTAP/DNA lipoplexes have shown to adsorb vitronectin which mediated receptor-dependent uptake by tumor cells expressing α_V_β_3_ integrins [265]. Patisiran is another prominent example for targeted transport to hepatocytes, mediated by coating with endogenous ApoE [104, 137]. Manipulation of the interaction with receptors *in vivo* and therefore targeted delivery to other cell types could be achieved by an altered protein corona as a consequence of exchanging certain lipid components of the Patisiran formulation [146]. Additionally, Saunders *et al*. used “nanoprimers” administered shortly before injection of therapeutic LNPs that were taken up by cells of the hepatic RES (Fig. 6B). By inhibiting KCs and LSECs, LNPs could be preferentially delivered to hepatocytes, the desired target site [266].

Based on these observations, methods which enable fast screening of broad libraries of nucleic acid carriers *in vivo* were developed [106-109]. By means of that, evaluation and characterization of predominant plasma proteins in the corona and tuning the biodistribution profile based on facile structural variations becomes feasible, pathing the way for improved targeted, cell-specific nucleic acid delivery *in vivo* (see Fig. 6C) [110, 142-145].

## Conclusion

Cell-specific delivery, especially for *in vivo* applications, remains a central challenge for the development of new nucleic acid therapeutics. Tremendous efforts were put into the optimization of existing delivery systems as well as in the development of new carriers due to numerous barriers that have to be overcome for efficient delivery and activity of the nucleic acid therapeutic. Receptor-mediated and chemical targeting strategies represent key approaches for targeted delivery and improved performance of synthetic carriers and have proven their ability for enhanced transfection efficacy in the desired cell type. Receptor-mediated nucleic acid delivery can be highly specific towards certain cell types (e.g., ASGPR) and even allows receptor-mediated transport across internal barriers (e.g., BBB). New, high-affinity ligands for specific delivery are discovered continuously and can be conjugated to synthetic carriers in numerous ways. Chemical targeting achieved by structural alterations of the particle components has also shown great potential for promoting cell-specific nucleic acid delivery. As a result, shifted biodistribution profiles were observed which enabled delivery to desired cells. In addition, new technologies as the barcoding method combined with high throughput processes could path the way for future applications, readily adjustable formulations and a deeper understanding of *in vivo* performance.
